# Management of ‘double eyelid ectropion’ using 5% hypertonic saline in an Indian newborn

**DOI:** 10.3205/oc000171

**Published:** 2020-11-26

**Authors:** Priti Bhoutekar, Dilip Kumre, Bhushan Uplanchiwar

**Affiliations:** 1 Department of Ophthalmology, Government Medical College, Chandrapur, Maharashtra, India

**Keywords:** congenital ectropion, double congenital ectropion, Indian newborn, conservative management, 5% hypertonic saline

## Abstract

**Objective:** This report describes a clinically rare case of congenital ectropion involving both upper lids in a one-day-old Indian newborn. We emphasize the importance of non-invasive conservative management with 5% hypertonic saline.

**Method:** Observational case report

**Result:** A term newborn presented to us on day 1 with bilateral upper lid ectropion or ‘double congenital ectropion’ noted since birth following an uneventful vaginal delivery. Examination revealed severe chemosis and prolapse of upper palpebral conjunctiva bilaterally. The repeated attempts to manually revert the eyelids in position failed. Otherwise, the eyes were normal. We started to treat the baby with topical hypertonic saline (5% sodium chloride), topical antibiotic, and topical lubricant frequently. Eye pads soaked in 5% hypertonic saline were also used. Following five days of treatment, the chemosis and ectropion resolved completely without recurrence.

**Conclusion:** We advocate non-invasive conservative management with 5% hypertonic saline soaked pads over the eyes along with topical antibiotic and lubricants. It should be the first line of treatment in all cases of congenital ectropion, before jumping to any aggressive invasive treatment like tarsorrhaphy, skin grafting etc., or unnecessary referral.

## Introduction

Congenital eyelid ectropion is a rare condition which usually presents at birth, but late-onset cases are also noted. It was first reported in 1896 by Adams, who used the term ‘double congenital ectropion’ [1]. In this condition, the eyelids are completely turned out with severe chemosis, which accentuates when the child cries. It can be associated with infection, inflammation, and birth trauma. The treatment is largely conservative using eye pads soaked in hypertonic saline (5% sodium chloride), antibiotic, and lubricants. Sometimes, invasive procedures like tarsorrhaphy, medial and lateral canthoplasties, horizontal lid shortening, and skin grafting [2], [3], especially in non-responsive cases or cases with Down’s syndrome may be required. These procedures should be essentially reserved for rare circumstances, and treatment with hypertonic saline should be initiated by a primary health care professional avoiding unnecessary referral.

## Case description

**Figure 1:**
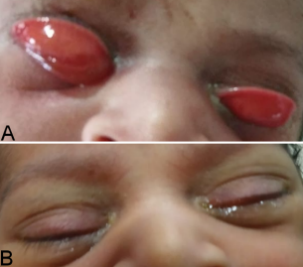
A) One-day-old neonate at presentation with bilateral ectropion, with the right eye showing a more severe chemosis than the left eye; B) On day 5 of conservative treatment, showing complete resolution of ectropion

Our case was a one-day-old term male neonate born to a 29-year-old Indian female. There was no significant antenatal history. The baby was born of an uneventful normal vaginal delivery and weighed 2.5 kg. He had eversion of both upper lids with chemosis, which progressively increased with crying (Figure 1 (Fig. 1)). Upper palpebral conjunctival prolapse with copious discharge was evident in both eyes. The attempts to manually invert the lids in position were not successful. Systemic examination of the baby was normal. On retracting the eyelids, the eyeballs appeared structurally normal. Posterior segment was also normal. Treatment with topical 5% hypertonic saline hourly as well as cotton gauzes soaked in 5% hypertonic saline were placed over the eyes constantly. Topical antibiotic (tobramycin 0.3%) was instilled three times a day along with lubricants every hour. The effect of the treatment was evident within 2–3 days with a reduction in chemosis and prolapse of the conjunctiva. Until the 4^th^ day of treatment, the eyelids were in a normal position and reverted again if the baby cried. Following five days of treatment, the eyelids and conjunctiva were almost normal with spontaneous eye-opening. There was no recurrence thereafter even if the baby cried. The baby was discharged on the 7^th^ day and was normal at subsequent follow-ups.

## Discussion

Congenital ectropion is an important entity not only because of its rarity, but also due to the panic it creates for parents and even health professionals if encountered for the first time. It usually presents at birth and is bilateral, though late-onset and unilateral cases are also reported. The exact incidence is unknown. Few sporadic cases have been reported after its first description by Adams [1], [4]. Association with Down’s syndrome [3], [4], collodion skin disease [5], and black infants [6] are documented in the literature. In the Indian population, only 4 cases have been reported till date, out of them one was associated with Down’s syndrome [7], another was a collodion baby [8], and two further reports were cases of congenital ectropion [9], [10]. We encountered this rare condition in a normal baby at a tertiary health care center in central India with no associated ocular or systemic abnormality.

The exact cause of ‘double congenital ectropion’ is unknown. A case report by Young (1954) described pathophysiology to be intrinsic to some eye disease which leads to inflammatory swelling and orbicularis spasm [11]. According to Young, during blepharospasm, if the conjunctiva protrudes out, it becomes strangulated at the lid margin which obstructs the venous return, which in turn increases the conjunctival chemosis leading to everted lids. Prolonged labor, birth trauma, or pre-existing vaginitis in the mother can cause infection or inflammation of the neonate’s conjunctiva.

Treatment is largely conservative, which if started promptly gives complete recovery from this condition. Topical antibiotic eye drops and frequent lubrication are required. However, we would like to emphasize the use of hypertonic saline (sodium hydrochloride 5%) eye drops and patching the eyes with gauzes soaked in the hypertonic saline solution.

The principle behind it appears to be ‘osmosis’, i.e. the hypertonic saline pulls out the fluid from the oedematous conjunctiva and thus drastically reduces chemosis. Based on the same principle, hypertonic sodium chloride ointment is used to reduce corneal oedema. We recommend the use of hyperosmolar saline patches as the first line of treatment since it is very effective and safe. The major side effects are unknown apart from mild allergic reactions, burning sensation, or irritation. In babies, an inappropriate increase in congestion of the eye should be taken cautiously and treated with antibiotics; if non-responsive, withholding hypertonic saline may be considered. Usually with this conservative approach, eyelids return to a normal position in 1–2 weeks without any after effects. Surgical intervention is needed in rare instances like non-response to conservative management, delay in conservative treatment leading to irreversible changes in the eyelids, Down’s syndrome, ichthyosis, etc. Parents need to be counseled and reassured regarding the benign nature of this condition and its complete recovery on topical medications as they can have anxiety, depression, and embarrassment. The non-surgical approach gives good anatomical and cosmetic outcomes without any sequelae if started appropriately and promptly.

## Conclusion

‘Double congenital ectropion’ is a rare condition having an alarming presentation but a benign course and excellent cosmetic outcomes after timely conservative treatment. We believe the use of 5% hypertonic saline-soaked eye pads over the eyes to be the most effective remedy. Primary health care providers – be it obstetrician, pediatrician, neonatologist or general ophthalmologist – should be aware of this condition to avoid any delay in initiating treatment and prevent any unnecessary referrals.

## Notes

### Informed consent

Informed consent has been obtained from the patient’s parents for the publication of this case report.

### Competing interests

The authors declare that they have no competing interests.
